# Melatonin Activates Anti-Inflammatory Features in Microglia in a Multicellular Context: Evidence from Organotypic Brain Slices and HMC3 Cells

**DOI:** 10.3390/biom13020373

**Published:** 2023-02-16

**Authors:** Sara Merlo, Grazia Ilaria Caruso, Dhwani Sunil Korde, Alla Khodorovska, Christian Humpel, Maria Angela Sortino

**Affiliations:** 1 Department of Biomedical and Biotechnological Sciences, Section of Pharmacology, University of Catania, 95123 Catania, Italy; 2 Laboratory of Psychiatry and Experimental Alzheimer’s Research, Medical University of Innsbruck, A-6020 Innsbruck, Austria; 3 PhD Program in Biotechnologies, University of Catania, 95123 Catania, Italy; 4 Department of Histology, Cytology and Embryology, Bukovinian State Medical University, Teatralnaya Square, 2, 58002 Chernivtsi, Ukraine

**Keywords:** organotypic brain slices, hippocampus, microglia, inflammation, melatonin, SIRT1, BDNF, HMC3 cells

## Abstract

Melatonin (MEL) is a neurohormone endowed with neuroprotective activity, exerted both directly on neuronal cells and indirectly through modulation of responsive glial cells. In particular, MEL’s effects on microglia are receptor-mediated and in part dependent on SIRT1 activation. In the present study, we exploited the highly preserved cytoarchitecture of organotypic brain cultures (OC) to explore the effects of MEL on hippocampal microglia in a 3D context as compared to a single cell type context represented by the human HMC3 cell line. We first evaluated the expression of MEL receptor MT1 and SIRT1 and then investigated MEL action against an inflammatory stimulation with LPS: OCs were cultured for a total of 2 weeks and during this time exposed to 0.1 μg/mL of LPS for 24 h either on day 1 (LPS 1°) or on day 11 (LPS 11°). MEL was added immediately after plating and kept for the entire experiment. Under these conditions, both MEL and LPS induced amoeboid microglia. However, the same round phenotype matched different polarization features. LPS increased the number of nuclear-NF-kB+ round cells and MEL alone or in combination with LPS increased BDNF+ round microglia. In addition, MEL contrasted LPS effects on NF-kB expression. Data from HMC3 microglia confirmed MEL’s anti-inflammatory effects against LPS in terms of CASP1 induction and BDNF release, identifying SIRT1 as a mediator. However, no effects were evident for MEL alone on HMC3 microglia. Overall, our results point to the importance of the multicellular context for full MEL activity, especially in a preventive view, and support the use of OCs as a favorable model to explore inflammatory responses.

## 1. Introduction

Melatonin (MEL) is a neurohormone primarily released by the pineal gland with a central role in circadian rhythm regulation [1]. Interestingly, MEL has gained attention for its several other beneficial activities, including anti-inflammatory [2], anti-oxidant [3] and anti-apoptotic [4] actions. These, together with MEL’s highly favorable safety profile and its permeability through the blood brain barrier [5,6], point to a relevant therapeutic potential against neurodegenerative conditions [7,8,9]. MEL acts on selective G-protein coupled receptors located at the cell surface, with two known isoforms, MT1 and MT2 [10,11,12]. Intra-nuclear ROR/RZR receptors [13,14] and a quinone reductase-2/MT3 mitochondrial receptor have been also described [15].

The beneficial effects of melatonin have been shown in pre-clinical and/or clinical studies for several neurodegenerative conditions triggered by insults such as trauma [16,17], hypoxia/ischemia [18,19], infections [20] or toxins [21] as well as for neurological disorders, such as Alzheimer’s disease [22,23,24,25], Parkinson’s disease [26,27] and amyotrophic lateral sclerosis [28]. A shared feature among these pathologies is neuroinflammation, a complex process mediated primarily by microglia and astrocytes [29]. In particular, microglia have been shown to activate readily in response to changes in the microenvironment and promote the orchestrated release of pro- and anti-inflammatory factors. Key signaling in promoting inflammation involves interleukin (IL)-1β, IL-6 and tumor necrosis factor (TNF)-α, while brain-derived neurotrophic factor (BDNF), IL-4 and IL-10 play a role in reparative activation. Acute inflammatory events prompt the removal of toxic debris and protein aggregates, supporting tissue repair and thus play a beneficial role in the restoration of homeostasis [30]. In agreement, the contribution of microglia to protective compensatory responses against toxic insults has been widely demonstrated [31,32,33]. However, when pro-inflammatory signaling cannot be timely resolved, inflammation can chronicize and turn into the harmful driver of a pathologic condition [34]. In general, anti- and pro-inflammatory microglia have been identified as two distinct phenotypes with opposite polarization and termed “M2” and “M1”, respectively [35,36]. The appropriateness of these labels has been extensively debated and they are not unanimously accepted on the account of microglia falling within a continuum of different phenotypes rather than in two static extremes [37,38]. Nevertheless, such nomenclature is still widely used to simplify the distinction between the prominence of protective versus detrimental features in microglia.

The possibility to modulate microglial activation to enhance its early protective functions or halt its harmful polarization over time is an intriguing strategy pursued by scientists in the last few years. In this context, MEL has been shown to activate the Silent Information Regulator 1 (SIRT1), a protective NAD+-dependent deacetylase, which mediates many of MEL’s functions [19,39,40,41]. SIRT1 is known to modulate gene transcription by deacetylation of histones and transcription factors, accounting for the largely demonstrated neuroprotective functions of the enzyme [42,43,44]. We confirmed the existence of a MEL-SIRT1-BDNF axis that induced anti-inflammatory effects in microglia of both murine [45] and human origin [46].

On these premises, we now intended to evaluate microglial responses to melatonin in a complex network of cell-to-cell signaling represented by the organotypic model. We explored the effects of MEL alone or under different settings of inflammatory stimulation with lipopolysaccharide (LPS) on microglia, in terms of morphological changes and expression of selective polarization markers. Additionally, in order to compare the effects of MEL on microglia devoid of input signaling from other cell types under these same experimental conditions, we performed a parallel study on the HMC3 human microglial cell line.

## 2. Materials and Method

### 2.1. Drugs

Melatonin (Santa Cruz Biotechnologies, Santa Cruz, CA, USA) was dissolved in dimethylsulfoxide (DMSO, Merck KGaA, Darmstadt, Germany) as a 10 mM stock and Lipopolysaccharide (LPS; Merck) was dissolved in water as 1 mg/mL stock. Both were subsequently diluted in culture medium for experiments.

### 2.2. Organotypic Brain Cultures (OCs)

Organotypic brain slices of the hippocampus (HC) were prepared from postnatal day 8–10 C57BL/6 wildtype mouse pups. All experiments conformed to Austrian guidelines on the ethical use of animals and were in line with the reduce, refine and replace (3Rs) rule as all efforts were made to reduce the number of animals and their suffering. At least 3 independent experiments were performed using 3–4 slices per group.

Vibrosections were generated under sterile conditions as described in detail [47]. After decapitation, brains were dissected, stuck onto the chuck of a water-cooled vibratome (Leica VT1000A, Biosystems, Nussloch, Germany) and placed near a sharp blade. Coronal 150 μm-thick vibrosections were cut and collected in sterile media. Thereafter, brain slices were carefully transferred onto a sterile 0.4-μm pore membrane (JHWP02500, Merck), which was placed into a 0.4-μm pore cell culture insert (PICM03050, Merck) within a 6-well plate (Greiner Bio-One, Kremsmünster, Austria). Each well contained 1 mL of sterile-filtered culture medium supplemented with 50% MEM/HEPES (ThermoFisher Scientific, Waltham, MA, USA), 25% heat-inactivated horse serum (HS; ThermoFisher), 25% Hanks’ solution (Thermofisher), 2 mM NaHCO_3_ (Merck), 6.5 mg/mL glucose (Merck), and 2 mM glutamine (Merck), at pH 7.2. The brain slices were cultured at 37 °C with 5% carbon dioxide (CO_2_) for 2 weeks and the culture medium was changed once a week. Brain slices were incubated without (control) or with 100 ng/mL LPS, 1 μM melatonin or a combination of both. At the end of the experiment, vibrosections were fixed for 3 h at 4 °C in 4% paraformaldehyde (PFA, Merck) and then stored at 4 °C in PBS until use.

### 2.3. Cell Cultures

The HMC3 human microglial cell line was purchased from the American Type Culture Collection (ATCC, LGC Standards, Manassas, VA, USA; Cat. No. CRL-3304). Cells were cultured in Eagle’s Minimum Essential Medium (EMEM; Merck; Cat. No. M4655) with the addition of 10% fetal bovine serum (FBS; Cat. No. 10500-064; ThermoFisher), sodium pyruvate (1×; ThermoFisher; Cat. No. 11360-070), non-essential amino acids (1×; Cat. No. 11140-035; ThermoFisher) and penicillin (100 U/mL)/streptomycin (100 μg/mL; ThermoFisher) at 37 °C with 5% CO_2_. Based on experimental plans, cells were plated with the following densities: 600 k cells/well in 6-well plates, 400 k cells/well in 12-well plates, 15 k cells/well in 96-well plates (Falcon, Milan, Italy).

### 2.4. Immunohistochemistry and Cell Counts on OCs

Immunohistochemistry was performed as previously reported in [47]. The fixed slices were incubated in 0.1% Triton-PBS (T-PBS) for 30 min at room temperature (RT), shaking. Sections were treated with PBS/1% H_2_O_2_/20% methanol to quench endogenous peroxidases. After that, slices were washed 3 × 3 min with PBS and subsequently blocked in T-PBS/20% HS/0.2% bovine serum albumin (BSA, Serva, Heidelberg, Germany) for 30 min at RT while shaking. For the detection by horseradish peroxidase- 3,3′-diaminobenzidine (HRP-DAB) method, slices were incubated with the following primary antibodies, diluted in T-PBS/0.2% BSA for 48 h at RT: rabbit anti-Iba1 (1:500; FUJIFILM Wako Chemicals, Richmond, VA, USA; Cat. No. 019-19741), rabbit anti-SIRT1 (1:250; ThermoFisher, Cat. No. PA5120542) or rabbit anti-melatonin receptor 1A (MT1A; 1:600; ThermoFisher, Cat. No. PA577490). The sections were then washed and incubated with the biotinylated secondary anti-rabbit antibody (1:200; Vector Laboratories, Vienna, Austria) in T-PBS/0.2% BSA for 1 h at RT while shaking. Following secondary antibody incubation, sections were washed 3 × 3 min with PBS and then incubated in avidin-biotin complex solution (Elite ABC kit, Vector) for 1 h at RT, shaking. Finally, the sections were washed with 50 mM Tris-buffered saline (TBS) and then incubated in 0.5 mg/mL DAB (Merck)/TBS/0.003% H_2_O_2_ at RT in the dark, until a signal was detected. Once DAB staining was visible, the reaction was stopped by adding TBS to the sections. The brain slices were washed again with PBS before being mounted on glass slides with Mowiol (Carl Roth, Karlsruhe, Germany). The staining was visualized with an Olympus BX61 microscope (Olympus Corporation, Shinjuku City, Tokyo, Japan) and Openlab software 5.5.0 (Improvision, Forchheim, Germany).

For fluorescent detection, the following primary antibodies were used: mouse anti-BDNF (1:200; ThermoFisher; Cat. No. MA5-34960), rabbit anti-Iba1 (1:700; Novus Biologicals, Englewood, CO, USA, Cat. No. NBP2-19019), mouse anti-NF-kB (1:400; Cell Signaling Technology, Danvers, MA, USA, Cat. No. 6956S). Slices were then washed and exposed to appropriate secondary antibodies for 1 h at RT while shaking, as follows: AlexaFluor (AF) 546-conjugated anti-rabbit (1:200; ThermoFisher) and AF488-conjugated anti-mouse (1:350; ThermoFisher), diluted in T-PBS/0.2% BSA. After washing, slides were mounted with 4′,6-diamidino-2-phenylindole (DAPI)-containing mounting solution (Merck; Cat. No. F6057). Digital images were captured at 20× magnification with a Zeiss Observer.Z1 microscope equipped with the Apotome.2 acquisition system (Zeiss, Oberkochen, Germany). Exposure was optimized independently for each image and no quantification of brightness was carried out. Five fields covering the whole HC were taken on both sides. The number of microglial cells/mm^2^ was determined by manual cell count using the ImageJ software (https://imagej.nih.gov/ij/download/, first access on 24 November 2016) [48].

### 2.5. Immunocytochemistry and Cell Counts on HMC3 Cells

Cells were fixed with InsideFix (Miltenyi, Bologna, Italy; Cat. No. 130-090-477) and incubated with the primary antibody rabbit anti-SIRT1 (1:80; Abcam, Cambridge, United Kingdom; Cat. No. ab189494) overnight followed by the secondary antibody AF488 plus-conjugated anti-rabbit (1:350; ThermoFisher) for 1 h at RT, shaking. Both antibodies were diluted in InsidePerm solution (Miltenyi; Cat. No. 130-090-477). Slides were mounted with DAPI-containing mounting solution (Merck) and digital captions were generated with a Zeiss Observer.Z1 microscope equipped with the Apotome.2 acquisition system (Zeiss). Exposure was optimized independently for each image and no quantification of brightness was carried out. The number of immunopositive cells with nuclear SIRT1 was determined by cell counting in at least five randomly selected fields/well using ImageJ software.

### 2.6. Quantitative Real-Time Polymerase Chain Reaction (qRT-PCR)

After cell collection, total RNA was extracted using the RNeasy Plus Mini Kit (Qiagen, Milan, Italy). RNA concentration was detected with Nanodrop spectrophotometer ND-1000 (ThermoFisher) and 2 µg of RNA were reverse transcribed using Superscript-VILO kit (ThermoFisher) according to the manufacturer’s instructions. qRT-PCR was performed on a 1:100 dilution of the reverse transcription reaction per sample, using the Rotor-Gene Q and Qiagen QuantiNova SYBR Green Real Time-PCR Kit. Primers used were Hs_BDNF_1_SG QuantiTect Primer Assay (QT00235368) and Hs_RPLP0_1_SG QuantiTect Primer Assay (QT00075012), both from Qiagen. RPLP0 was used as the endogenous control for normalization. Melting curve analysis confirmed the specificity of the amplified products. Data were analyzed applying the ∆∆Ct method and expressed as fold change versus control.

### 2.7. Western Blot

Organotypic cultures were scraped off membranes and collected into 1.5 mL-test tubes, lysed in 80–120 µL of PBS containing EDTA-free protease inhibitor cocktail (Merck; Cat. No. P-8340). HMC3 cells were collected and lysed in ice-cold radioimmunoprecipitation assay (RIPA; Merck; Cat. No. R0278) supplemented with anti-protease and anti-phosphatase cocktails (Merck; P2850).

Samples were sonicated and centrifuged at high speed for 5 min at 4 °C and protein concentration was determined by a Bradford reagent (Merck) at 595 nm absorbance, measured with Varioskan^TM^ Flash Multimode Reader (ThermoFisher). Equal amounts of protein extracts were loaded on pre-cast “any-kDa” or 4–20% gradient gels (Bio-Rad, Hercules, CA, USA) in sodium dodecyl sulfate-poly-acrylamide gel electrophoresis (SDS-PAGE), followed by transfer to nitrocellulose membranes (Hybond ECL; Amersham Biosciences Europe GmbH, Milan, Italy) through a Transblot semidry transfer cell (Bio-Rad) for 70 min. Membranes were blocked with a Blocker FL Fluorescent Blocking buffer (ThermoFisher) and incubated with primary antibodies rabbit anti-SIRT1 (1:1000; Abcam; Cat. No. ab189494), rabbit anti-Iba1 (1:700; Novus) and rabbit anti-β-actin (1:3000; Merck; Cat. No. A2066) for 24–48 h at 4 °C. After that, the incubation with secondary antibody IRDye^®^ 800CW-conjugated anti-rabbit (1:1000; LI-COR Biosciences, Lincoln, Nebraska USA) was conducted for 1 h at RT. iBright FL1500 Imaging System (ThermoFisher) was used to detect bands and ImageJ software to analyze band intensity.

### 2.8. Enzyme-Linked Immunosorbent Assay (ELISA)

Released BDNF was measured with the Biosensis^®^ BDNF Rapid^TM^ ELISA kit (Biosensis Pty Ltd., Thebarton, SA, Australia; Cat. No. BEK-2211-1P), according to the manufacturer’s instructions. Absorbance at 450 nm was measured with Varioskan^TM^ Flash Multimode Reader (ThermoFisher).

### 2.9. Caspase 1 Assay

Selective caspase 1 proteolytic activity was assessed using the fluorogenic substrate Ac-VAD-AFC (S. Cruz), according to a previously published protocol [49]. Briefly, HMC3 cells were collected and lysed in ice-cold RIPA buffer devoid of protease inhibitors. Protein concentration was determined with Bradford reagent and 100–200 μg/sample were diluted with RIPA to a final volume of 60 μL. These were added to 60 μL of caspase activity buffer (100 mM Hepes pH = 7.5; 10% glycerol; 10 mM dithiothreitol) containing 100 μM of Ac-VAD-AFC. The mix was incubated for 1 h at 37 °C. Absorbance was determined at λEx/λEm = 400/505 nm using a Varioskan^TM^ Flash Multimode Reader (ThermoFisher).

### 2.10. Statistical Analysis

All data were from three or more independent experiments. Experimental values are presented as the mean ± SEM. Statistical analysis was performed by Student’s *t*-test, when two conditions were compared, or one-way ANOVA followed by Fisher LSD post hoc test, as appropriate, when multiple comparisons were made. *p* ≤ 0.05 was set as the criterion for statistical significance. All statistical analyses were carried out using GraphPad Prism Software (GraphPad Software, San Diego, CA, USA).

## 3. Results

### 3.1. Organotypic Slices Express the MT1 Receptor and Respond to Melatonin by Upregulation of SIRT1 and Iba1

**Figure 1:**
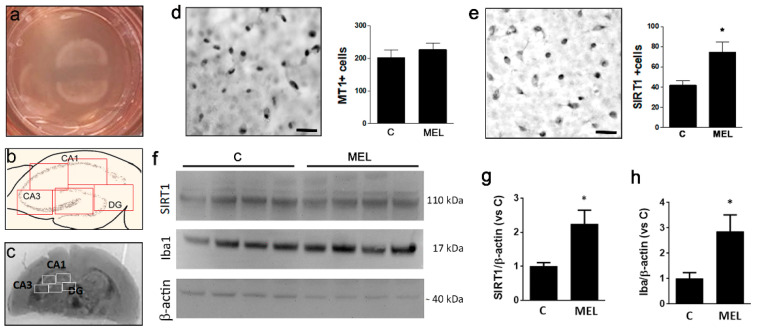
Overview of organotypic brain cultures (OCs). In (**a**), OCs (150 μm) prepared from 8–10 day postnatal wild-type mice after culturing for 2 weeks on an insert at the liquid/air interface. The two hippocampal formations (HC) were visible in each slice (**c**). Five areas, indicated by the boxes in (**b**,**c**), were imaged to cover the entire HC and analyzed for the number of melatonin receptor 1 (MT1)- (**d**) or SIRT1-positive (**e**) cells in control condition (C) or after exposure to melatonin (MEL) for 14 days. The protein levels of SIRT1 (**f**,**g**) and Iba1 (**f**,**h**) were detected by Western blot. Representative images are shown. Scale bars (**d**,**e**) = 100 μm. Data are the mean ± SEM of at least 3 experiments. * *p* < 0.05 vs. C by Student’s *t*-test for significance.

Organotypic slices of 150 μm were cut from the brain portion containing the hippocampus (HC) and cultured for 2 weeks on a membrane insert, at the interface between air and liquid (Figure 1a). The hippocampal cornus ammonis (CA)1, CA3 and dentate gyrus (DG) areas (as depicted in Figure 1b) were clearly visible (Figure 1c). For cell counts, the whole HC was analyzed by capturing 5 images covering all areas, as indicated by the boxes in Figure 1b,c.

The expression of MT1, the main MT in the CNS and centrally involved in neuroprotective effects, was confirmed in OCs by immunostaining (Figure 1d). Exposure of OCs to MEL for 2 weeks starting from day 1 after culture did not affect the number of MT1+ cells (Figure 1d), thereby excluding any feedback mechanism modulating sensitivity to the hormone. The expression of SIRT1 was confirmed in the HC by immunostaining and Western blot, and showed a significant increase both in the number of immunopositive cells (Figure 1e) and in protein levels (Figure 1f,g) following MEL treatment during the 2 weeks of culture. This result confirmed SIRT1 to be a target for melatonin signaling. Finally, selective microglial marker Iba1 was significantly increased in the whole brain slice following exposure to MEL, as by Western blot (Figure 1f,h).

### 3.2. Melatonin and LPS Activate Microglia into a Round Morphology

**Figure 2:**
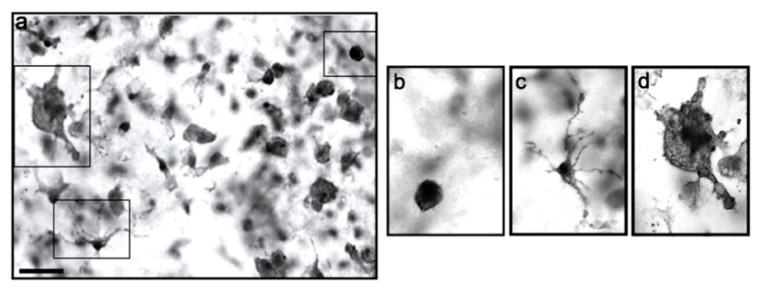
Microglia in the HC of OCs. Microglial cells were identified by selective labeling with anti-Iba1 and show a mixed population in terms of morphology (**a**). Microglia were mainly round-shaped (**b**) or highly ramified (**c**) though a few macrophage-like cells were also evident (**d**). Scale bar = 120 μm (**a**) and 60 μm (**b**–**d**).

Given the unexpected increase in Iba1 expression following MEL exposure, we decided to investigate this effect in more detail by immunohistochemical analysis, which allows the direct visualization of microglial cell distribution and morphology. Microglial Iba1 labeling revealed the presence of a heterogeneous population of cells in the area of the HC (Figure 2a). Round (Figure 2b) and ramified cells (Figure 2c) were prevalent, although a few macrophage-like shaped elements could also be detected (Figure 2d).

**Figure 3:**
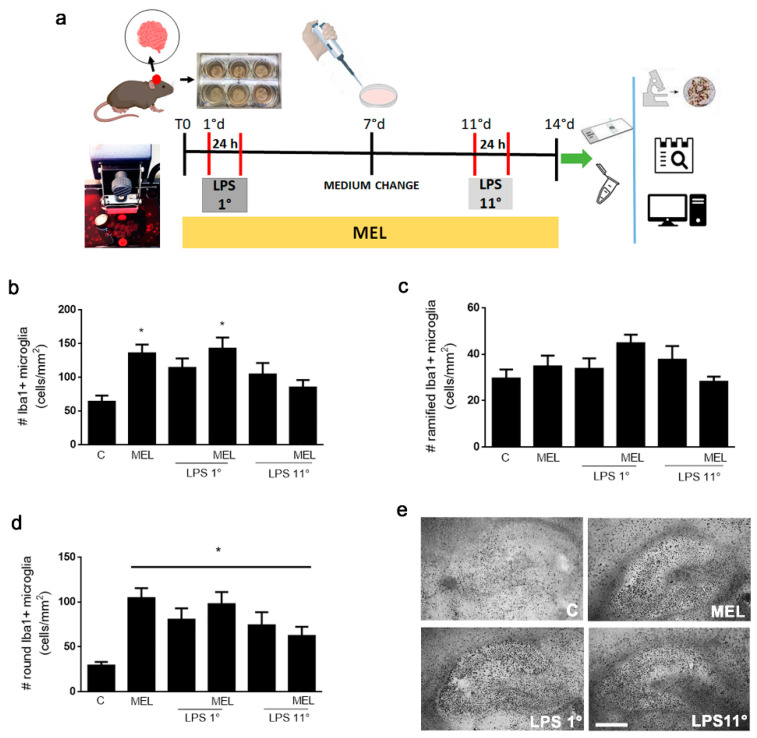
Morphological changes induced by MEL and LPS. As outlined in panel (**a**), brains were isolated and sectioned with a vibratome; the slices were transferred to membrane inserts into a culture plate and exposed to LPS (0.1 μg/mL) for 24 h, either on the first day after culture (LPS 1°) or on the eleventh day (LPS 11°) and further cultured for a total of 14 days in vitro. Melatonin (MEL; 1 μM) was added to OCs for 14 days, with or without LPS. Untreated condition is indicated as C. Microglial cells were immunolabeled with Iba1, counted and graphed as the total (**b**), ramified (**c**) or round (**d**) cell numbers (#). In (**e**), representative images of the distribution of Iba1-positive microglia in the whole hippocampal area. Scale bar = 580 μm. Data are means ± SEM with *n* ≥ 3. * *p* ≤ 0.05 vs. C by one way-ANOVA followed by Fisher LSD test post hoc for significance.

Organotypic cultures were treated according to the protocol illustrated in Figure 3a. The slices were pulsed with LPS (0.1 μg/mL) for 24 h either the day after dissection or on day 11, then cultured until day 14. When present, MEL (1 μM) was added to the slices starting on the day of dissection and until the end of the experiment. Control slices were cultured in medium without the addition of any drug. The two protocols allowed to test the effects of MEL in a setting of recovery from a toxic insult or before exposure to it. Microglial cells were labeled with anti-Iba1 antibody and the total number of cells (Figure 3b) was manually counted and classified as “ramified” (Figure 3c) or “round” (Figure 3d), based on morphology. As shown in Figure 3b, the total number of microglial cells showed a trend towards an increase, which reached significance in the presence of MEL alone or in combination with LPS added on day 1 (MEL + LPS 1°). While the number of ramified cells (Figure 3c) was not significantly affected by treatment, the round cell population (Figure 3d) was significantly increased by all treatment conditions. Representative images of Iba1 labeling showing the hippocampal distribution of positive cells are reported in Figure 3e.

### 3.3. Melatonin Induces an Anti-Inflammatory Signaling and Is Able to Contrast the LPS Pro-Inflammatory Trigger

**Figure 4:**
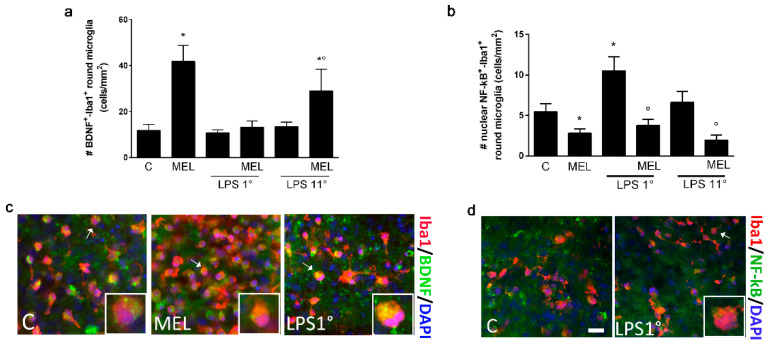
Differential polarization of round microglia. OCs were exposed to LPS (0.1 μg/mL) for 24 h, either on the first day after culture (LPS 1°) or on the eleventh day (LPS 11°) and further cultured for a total of 14 days in vitro. Melatonin (MEL; 1 μM) was added to OCs for 14 days, with or without LPS. Microglial cells were co-immunolabeled for Iba1+ with either BDNF or NF-kB. Cell counts of double-positive round cells for each group are reported in (**a**,**b**). Representative pictures are shown in (**c**) (Iba1^+^/red and BDNF^+^/green) and (**d**) (Iba1^+^/red and NF-kB^+^/green). Nuclei are counterstained with DAPI (blue, (**c**,**d**)). Insets show a detail of the single cells indicated by the arrows, magnified 2.5×. Scale bar = 25 μm. Data are mean ± SEM with *n* = 3. * *p* < 0.05 vs. C (over bars in (**a**,**b**)) and ° *p* < 0.05 vs. respective LPS (over bars in (**a**,**b**)) by one-way ANOVA followed by Fisher’s LSD test for significance.

Since an amoeboid morphology can be correlated with both a pro- and anti-inflammatory polarization, we subsequently performed selective analysis of two representative markers for each condition. In particular, we chose BDNF (for “M2-like”) and nuclear NF-kB (for “M1-like”), each labeled in double immunostainings with Iba1 (Figure 4c,d). Counts were performed selecting double-positive cells among the round population, co-expressing Iba1 with each polarization marker. Results pointed to a differential phenotype acquisition for round cells exposed to different treatments. MEL induced the number of BDNF^+^ cells (Figure 4a) while reducing the number of NF-kB^+^ microglia (Figure 4b). LPS given at day 1 followed by a recovery incubation did not modify BDNF expression (Figure 4a), but the number of cells with nuclear NF-kB was increased (Figure 4b). In this condition, MEL was not able to increase BDNF^+^ cells but it contrasted the nuclear expression of NF-kB. When OCs were exposed to LPS on day 11, neither BDNF nor NF-kB expression were significantly affected, but MEL was able to skew round microglia towards a BDNF-expressing phenotype, with significantly less NF-kB^+^ cells compared to LPS 11° alone (Figure 4a,b). These results confirmed the existence of different activated states of microglia, sharing a similar round morphology but opposite in their polarization. Importantly, MEL was confirmed to enhance an anti-inflammatory signaling and to suppress pro-inflammatory activation in the presence of LPS.

### 3.4. In HMC3 Microglia MEL Retains Anti-Inflammatory Activity against LPS but Is Devoid of Effects per Se

**Figure 5:**
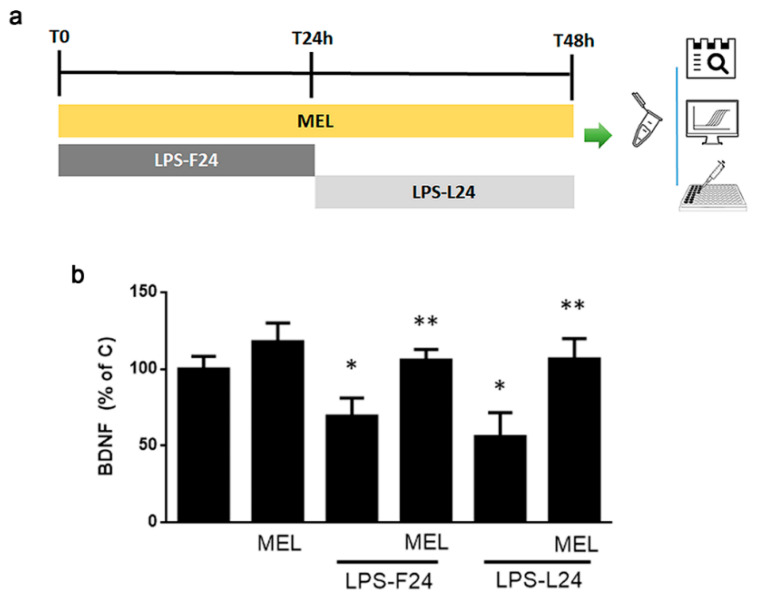
Polarization of HMC3 microglia. In (**a**), the experimental paradigm used is reported. MEL (1 μM) was always added for 48 h. When in combination with LPS (1 μg/mL), two different settings were used: LPS was added for the first 24 h, followed by removal and further incubation for 24 h (LPS-F24); alternatively, cells were incubated for 24 h prior to addition of LPS for the last 24 h (LPS-L24). In (**b**), BDNF release evaluated by ELISA. Data are means ± SEM of *n* = 3 experiments. * *p* < 0.05 vs. C and ** *p* < 0.05 vs. respective LPS-treated by one-way ANOVA followed by Fisher-LSD test for significance.

In order to evaluate the significance of the multicellular context in MEL’s ability to modulate microglial polarization, we carried out a parallel in vitro study using the human microglial cell line HMC3. We adopted an experimental paradigm that would mimic the exposure to MEL for a prolonged time (48 h), with a shorter pulse (24 h) of LPS either in the beginning, followed by a recovery time (LPS-F24), or in the last 24 h of treatment (LPS-L24). Cells were then collected to assess polarization markers through different approaches, according to the protocol illustrated in Figure 5a.

Microglial polarization was evaluated by analysis of BDNF mRNA expression by RT-PCR and release by ELISA. BDNF mRNA expression was not affected by LPS exposure (not shown). BDNF protein release was significantly reduced by LPS in both conditions, an effect always prevented by MEL; however, it lacked any effect when added alone (Figure 5b).

**Figure 6:**
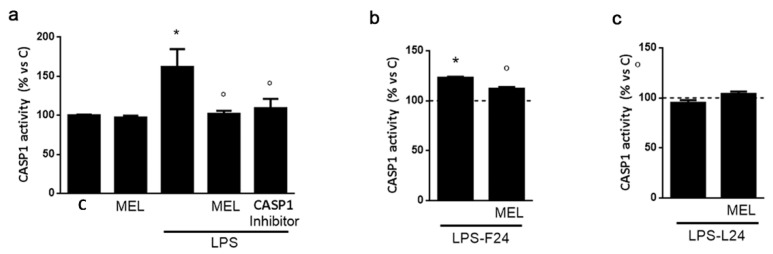
Caspase 1 (CASP1) activity assay in HMC3 microglia. Activation of CASP1 was assessed by incubation with the fluorogenic substrate Ac-VAD-AFC. In (**a**), LPS (1 μg/mL), MEL (1 μM) and CASP1 inhibitor (10 μM) were added for 48 h. In (**b**), MEL (1 μM) was added for 48 h alone or in combination with LPS (1 μg/mL), which was present only during the first 24 h, followed by removal and further incubation for 24 h (LPS-F24). In (**c**), MEL (1 μM) was added for 48 h alone or in combination with LPS (1 μg/mL), which was added to the cells only during the last 24 h (LPS-L24). In (**b**,**c**), dotted lines indicate control untreated condition (C). Data are mean ± SEM of 3 independent experiments. * *p* < 0.05 vs. C and ° *p* < 0.05 vs. respective LPS by one-way ANOVA followed by Fisher’s LSD post hoc test for significance.

Next, we investigated the activation of caspase 1, the major catalytic component of the inflammasome, responsible for proteolytic activation of several pro-inflammatory factors. Using a fluorogenic substrate detectable upon selective cleavage by caspase 1, we were able to show that 48 h of exposure to LPS (1 μg/mL) significantly activated the protease, an effect prevented by MEL (1 μM; Figure 6a), which did not have any effect per se. Caspase 1 selective inhibitor (10 μg/mL) was used as an internal control and confirmed a preventive effect on cleavage of the substrate (Figure 6a). Applying the protocol illustrated in Figure 5a, we found that while LPS given in the first 24 h significantly increased caspase 1 activity (Figure 6b), with a preventive effect by MEL exposure to LPS in the last 24 h of treatment did not exert any effect (Figure 6c).

**Figure 7:**
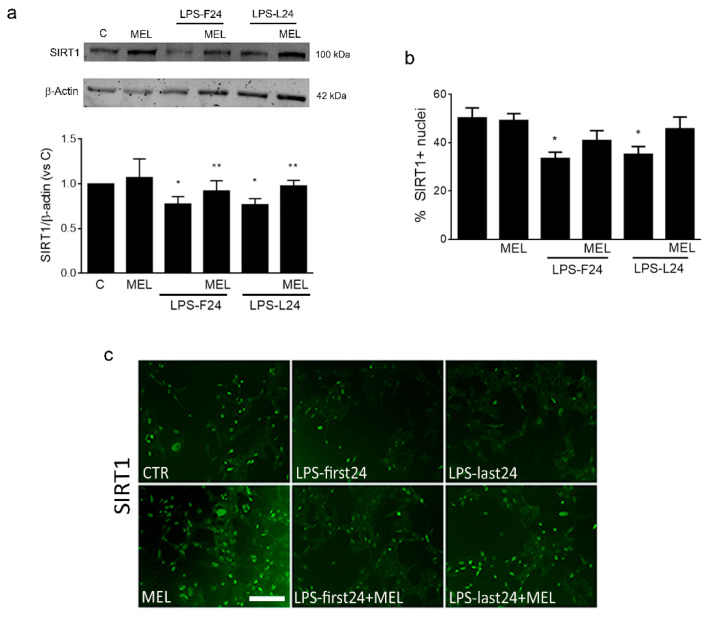
MEL’s effects on SIRT1 expression in HMC3 cells under inflammation. MEL (1 μM) was always added for 48 h. When in combination with LPS (1 μg/mL), two different settings were used: LPS was added for the first 24 h, followed by removal and further incubation for 24 h (LPS-F24); alternatively, cells were incubated for 24 h prior to addition of LPS for the last 24 h (LPS-L24). In (**a**), total SIRT1 expression was evaluated by Western blot analysis and a representative blot is shown. The percentage of cells with nuclear SIRT1 was then determined by immunolabeling, followed by cell count. Results are graphed in (**b**) and representative images of immunostaining (green) are shown in (**c**). Scale bar = 80 μm. Data are mean ± SEM of 3 independent experiments. * *p* < 0.05 vs. C and ** *p* < 0.05 vs. corresponding LPS-treated, by one-way ANOVA followed by Fisher-LSD post hoc test for significance.

Finally, we explored the involvement of SIRT1 in MEL-mediated effects. MEL alone (1 μM for 48 h) did not modify SIRT1 expression but it was able to contrast the LPS-driven reduction of the protein, as by Western blot (Figure 7a). The same effects were confirmed by immunocytochemical analysis of nuclear SIRT1 (representative images shown in Figure 7c), although in this case MEL action against LPS did not reach statistical significance (Figure 7b).

## 4. Discussion

The present study was undertaken to assess how melatonin affected microglial activation/polarization in basal or inflammatory conditions, in a complex microenvironment represented by organotypic cultures. This ex vivo model is particularly valuable since it joins the advantages offered by in vitro cultures and in vivo models, i.e., an easier manipulation but with the preservation of an intercellular signaling network. Our attention was focused in particular on the HC, a region involved in neuronal plasticity and memory and thus especially at risk for neurodegeneration [50]. The two experimental protocols used were chosen with the aim of dissecting MEL’s anti-inflammatory potential both as a support in the recovery and as a preventive option against pro-inflammatory challenges. MEL deserves attention as a potential therapeutical agent in the CNS for its notable safety [5] and for the ability to cross the blood-brain barrier [51,52] associated with a variety of proven beneficial effects, deriving from its action on multiple targets [53,54,55]. Evidence from the literature, including our own work, clarified that microglia are a direct target for MEL action, with neuroprotective outcomes [45,56,57].

Firstly, we verified the general responsiveness of the OC model to MEL in terms of MT1 expression and SIRT1 upregulation. We showed that MT1 receptor is expressed in OCs in agreement with the involvement of this receptor subtype in the beneficial actions of MEL in the CNS, as shown in other models. Interestingly, both in a Huntington disease transgenic mouse model [58] and in a PD mouse model [59], MT1 was significantly reduced; in the latter the reduction of MT1 was linked to a greater sensitivity to LPS-induced neuroinflammation. Our work in this direction was only preliminary and future studies will be directed to a full functional examination of MT1’s role in our model.

The involvement of SIRT1 as a mediator of MEL effects has been well established and its importance lies within the several anti-inflammatory and anti-aging properties attributed to this deacetylase across species [60,61]. We thus examined the effects of MEL on SIRT1 levels and found a significant increase in OCs after 2 weeks of treatment. Data from the literature show that baseline induction of SIRT1 by MEL is not always detectable, likely due to different experimental settings and models. In our own previous work, we described a trend towards MEL-induced SIRT1 increase in sham-operated rats serving as controls in hypoxia-related experiments, but the effect did not reach significance [45]. In general, we can speculate that MEL-driven basal upregulation of SIRT1 can ameliorate the overall cellular health and predispose to a more efficient defensive response. This is in agreement with our recent data in the OC model, indicating improved viability of cholinergic neurons from the nucleus basalis of Meynert following MEL/SIRT1 increase [62].

Our interest was eventually directed to the selective effects of MEL on microglia, the main cell type devoted to CNS protection. Microglia act through a dynamic adaptation to their microenvironment that prompts changes in their morphology and signaling [63]. In this part of the study, we included an inflammatory stimulus and proposed a dual exposure protocol to assess MEL’s effects on OC microglia: (1) during a toxic pulse and for a given recovery time afterwards or (2) prior to the toxic pulse and maintained together with it. In parallel, we used a human microglial cell line to compare the effects of MEL on microglia devoid of input signaling from other cell types under a similar experimental paradigm.

Melatonin proved once again to be a potent activator of microglial anti-inflammatory signaling. In fact, microglia acquired a round phenotype, characterized by increased BDNF expression and reduced nuclear NF-kB localization. Notably, the number of ramified, quiescent cells was unaffected. This could be indicative of an increased proliferation of activated microglia, which has been previously reported in cell lines such as BV2 or N9 [64,65,66]. Alternatively, the observed increase could be linked to an induction of Iba1, known to be upregulated during microglial activation. Iba1 is a calcium-binding protein involved in membrane modifications that are necessary for microglial shape change and migration [67,68]. As expected, LPS similarly induced an activated phenotype but in this case associated with increased nuclear NF-kB, indicative of a pro-inflammatory polarization. MEL’s ability to prevent NF-kB activation was evident in the presence of LPS in both exposure paradigms, suggesting a protective effect against inflammation both after and before exposure to the toxic stimulus. However, the induction of BDNF was partially restored only when the LPS pulse was preceded by MEL exposure and not in a recovery phase after the LPS pulse.

The application of a similar paradigm of LPS stimulation/MEL exposure to microglial cells alone, using the HMC3 cell line, exerted similar effects to those observed with OCs regarding LPS inflammatory induction and MEL’s tendency to contrast it. In particular, caspase 1 activity was analyzed as an index of inflammation, as an upstream activator of interleukins and of the inflammasome [69,70] while SIRT1 was investigated for its established role in microglial BDNF upregulation and anti-inflammatory phenotype acquisition [45,46,56,71,72]. In both cases the data go in the same direction and prove that MEL was always able to prevent the effects under study. Accordingly, MEL prevents induction of several inflammatory mediators, such as COX2, iNOS, NF-kB, cytokines and interleukins in murine BV2 microglia [73,74,75]. In these studies, MEL was used at higher concentrations, likely involving different modes of action including anti-oxidant effects. In addition, SIRT1 is confirmed to be a key player in evoking anti-inflammatory features of microglia.

Altogether, results from HMC3 experiments suggested that microglial reactivity to LPS-induced inflammation and MEL’s ability to affect it are substantially independent of inputs from other cells. Curiously however, the intense stimulating effects observed by MEL alone were only present within the complex microenvironment of OCs and not on microglia only. This result points to the quite relevant conclusion that in the absence of inflammatory cues, melatonin-activated signaling is affected by other cell types that somehow act to reinforce it. This aspect supports the preventive value of MEL and surely deserves further investigations in the future. Our work additionally provides information on the timing of MEL action in respect to that of the insult in ex vivo and in vitro models, and points to an efficient effect of MEL both in preventive and therapeutic approaches.

In conclusion, through the exploitation of ex vivo and in vitro models, our study highlights the significance of the multicellular context for the full expression of MEL anti-inflammatory activity, pointing to a major role for its preventive action. Furthermore, our results support the use of OCs as a favorable model to explore inflammatory responses in different settings.
